# Multimetallic Nanoparticles as Alternative Antimicrobial Agents: Challenges and Perspectives

**DOI:** 10.3390/molecules26040912

**Published:** 2021-02-09

**Authors:** Nagaraj Basavegowda, Kwang-Hyun Baek

**Affiliations:** Department of Biotechnology, Yeungnam University, Gyeongsan, Gyeongbuk 38451, Korea; nagarajb2005@yahoo.co.in

**Keywords:** alternative antimicrobial materials, infectious diseases, multidrug resistance, multimetallic nanoparticles, synergistic effect

## Abstract

Recently, infectious diseases caused by bacterial pathogens have become a major cause of morbidity and mortality globally due to their resistance to multiple antibiotics. This has triggered initiatives to develop novel, alternative antimicrobial materials, which solve the issue of infection with multidrug-resistant bacteria. Nanotechnology using nanoscale materials, especially multimetallic nanoparticles (NPs), has attracted interest because of the favorable physicochemical properties of these materials, including antibacterial properties and excellent biocompatibility. Multimetallic NPs, particularly those formed by more than two metals, exhibit rich electronic, optical, and magnetic properties. Multimetallic NP properties, including size and shape, zeta potential, and large surface area, facilitate their efficient interaction with bacterial cell membranes, thereby inducing disruption, reactive oxygen species production, protein dysfunction, DNA damage, and killing potentiated by the host’s immune system. In this review, we summarize research progress on the synergistic effect of multimetallic NPs as alternative antimicrobial agents for treating severe bacterial infections. We highlight recent promising innovations of multimetallic NPs that help overcome antimicrobial resistance. These include insights into their properties, mode of action, the development of synthetic methods, and combinatorial therapies using bi- and trimetallic NPs with other existing antimicrobial agents.

## 1. Introduction

Pathogenic bacteria are abundant in the environment. They spread quickly and can easily cause adverse reactions, long-lasting health effects, or even death. Infection originating from the invasion of pathogens into the body is an acute threat to humans, causing diseases, such as pneumonia, gastritis, and sepsis, which can lead to tissue damage, organ failure, and death [1]. Antibiotics have immense benefits in the fight against a diverse range of pathogens. However, mutations are one strategy that bacteria employ to enhance their resistance to antibiotics, leading to the advent of a large number of multidrug-resistant (MDR) strains, which markedly lowers the therapeutic ability of antibiotics. There is increasing concern regarding the generation of antibiotic resistance, as bacteria vigorously persist to emerge with flexible countersteps against conventional antibiotics [2]. This is one of the most notable health-related matters of the 21st century [3]. Infectious diseases caused by MDR bacteria and the abundance of these bacteria have increased at an alarming rate, especially penicillin-resistant *Streptococcus pneumoniae*, methicillin-resistant *Staphylococcus aureus*, vancomycin-resistant *Enterococcus faecium*, ceftazidime-resistant *Klebsiella pneumonia* and *Escherichia coli*, fluoroquinolone-resistant *Pseudomonas aeruginosa*, and multi-antibiotic resistant *Acinetobacter baumannii* [4,5]. In addition, various foodborne pathogens associated with Gram-positive bacteria, such as *Bacillus cereus*, *Campylobacter jejuni*, *Clostridium perfringens*, *Clostridium botulinum*, *Cronobacter sakazakii*, *E. coli*, *Listeria monocytogenes*, *Salmonella enteritidis*, *Shigella dysenteriae*, *S. aureus*, *Vibrio furnissii*, and *Yersinia enterocolitica*, are causing a large number of diseases, with major effects on public health and safety [6].

The production of new antibiotics requires enormous economic and labor demands and is a time-consuming process. Hence, the development of alternative, unconventional strategies to treat infectious diseases has become highly advisable [7]. The combination of one antimicrobial agent with other antimicrobial agents has many advantages, such as increased biological activity, reduced adverse effects, and increased antimicrobial toxicity of the combined elements. In synergism, one antimicrobial agent influences the activity of the other, and they finally act more efficiently and effectively together due to their different mechanisms of individual action. This may be considered a new approach to solve the problem of bacterial resistance and reduced antimicrobial susceptibility. Thus far, many alternative strategies have been developed to combat MDR bacteria. Among them, several in vitro studies have confirmed the significant antimicrobial activities of combinations of essential oils/plant extracts, conventional antibiotics/plant extracts, and phytochemicals/antibiotics [8].

Nanotechnology has important commercial applications in the fields of biology and medicine, particularly in the areas of drug delivery, diagnosis, tissue engineering, imaging, and bacterial infections [9]. Nanomaterials have special properties owing to their small dimensions; electrical, mechanical, optical, and magnetic properties; thermal stability; and high surface-to-volume ratio [10]. Nanomaterials are considered favorable alternatives to antibiotics for controlling bacterial infections due to a diverse range of factors, such as their size, morphology, surface charge, stability, and concentration in the growth medium. The surface coatings of nanoparticles (NPs) play an important role in influencing the antimicrobial properties of nanomaterials [11,12]. Depending on the number of metals, metal and metal oxide NPs are divided into mono-, bi-, tri-, and quadrometallic types. Among these, bi-, tri-, and multimetallic NPs have attracted the greatest interest due to their enhanced catalytic properties and favorable characteristics compared with monometallic NPs [13]. Multimetallic NPs are novel materials that incorporate two or more metals to make alloys with different functionalities and tunable properties, such as catalytic and optical properties. Multimetallic NPs can be modified by controlling their structure, chemical composition, and morphology to achieve maximum synergistic performance [14]. The addition of one or more metals into the NPs is expected to bring combinatorial effects, such as alteration of the electron structure, deduction of the lattice distance, and improvements in the total electronic charge shift [15]. Therefore, the study of the multidimensional space is warranted.

Monometallic NPs possess only one type of metal with specific chemical and physical properties, such as Ag, Au, Zn, Pd, Cu, Ti, Si, Al, Se, and Mg, which have been used for their antimicrobial activity for centuries. Among these, Ag NPs are the most effective as they are able to kill both Gram-positive and Gram-negative bacteria, and they are even effective against drug-resistant species [16]. Moreover, metal oxide NPs, such as Ag_2_O, ZnO, CuO, TiO_2_, NiO, Fe_3_O_4_, α-Fe_2_O_3_, CaO, MgO, Al_2_O_3_, CeO_2_, Mn_3_O_4_, and ZrO_2_ NPs, have highly potent antibacterial effects against a wide spectrum of microorganisms [17]. Similarly, metal sulfide and metal–organic framework (MOF) nanomaterials, such as AgS-, FeS-, CdS-, and ZnS-MOFs and Mn-, Cu-, and Zn-based MOFs, have demonstrated antimicrobial activities [18]. Bimetallic NPs are formed via the integration of two different types of metal atoms to form a single nanometric material with varying structures, morphologies, and properties [19]. Bimetallic NPs can be tuned by selecting the appropriate metal precursors to achieve the desired shape, size, structure, and morphology according to the configuration of atoms, and this finally leads to the formation of alloy, core–shell, and aggregated nanoparticle types [13]. Bimetallic NPs, such as Ag/Au, Ag/Cu, Au/Pt, Au/Pd, Ag/Fe, Fe/Pt, Cu/Zn, Cu/Ni, Au/CuS, and Fe_3_S_4_/Ag NPs, have distinctive surface activities. In addition, bimetallic oxide NPs, such as MgO/ZnO, CuO/ZnO, and Fe_3_O_4_/ZnO NPs, due to tensile strain and synergism between the constituent metals, often exhibit unique antibacterial performance.

Similarly, trimetallic NPs are made from three different metals for lowering metal consumption, atomic ordering, and fine-tuning the size and morphology of these NPs. Trimetallic NPs exhibit higher catalytic selectivity/activity and efficiency in various applications, such as biomedical, antimicrobial, catalytic, active food packaging, and sensing applications. Moreover, owing to the presence of three metals, there are some possibilities for different structures and morphologies, such as core–shell, mixed structure, subcluster segregated, and multishell [20]. To change their catalytic performance, trimetallic NPs were further designated as alloys and intermetallic NPs by altering the atomic distribution and surface compositions of different metals [21]. Trimetallic NPs exhibit innovative physicochemical properties owing to their synergistic or multifunctional effects for diverse potential applications when compared with monometallic and bimetallic nanomaterials. However, to date, only monometallic, bimetallic, and very few trimetallic NPs have been reported for their antimicrobial effects.

Hence, the distinctive properties of nanomaterials provide a favorable environment for antibacterial therapies when compared with their bulk forms. Many inorganic and organic nanocomposites exhibit potential antibacterial properties with fast and sensitive bacterial detection. Nanocomposites are designed with targeted and sustained release mechanisms, environmental responsiveness, and combinatorial delivery systems for antibacterial therapies [22]. In particular, metal and metal oxide NPs, with nanotoxic mechanisms and collective modes of action, cause membrane damage and produce reactive oxygen species (ROS) that act against bacterial cells [23], which is why pathogens can barely develop resistance against them. The release of metal and metal oxide ions is the main mechanism responsible for the antimicrobial properties of nanocomposites. The current review aimed to highlight the most recent literature on mono-, bi-, tri-, and multimetallic NPs and their antimicrobial abilities. We discuss the importance, properties, fabrication methods, antimicrobial activities, and mechanisms of multimetallic NPs. Additionally, the synergistic effects, toxicity, and future prospects of multimetallic NPs are discussed. This study is expected to enhance our understanding of the development of multimetallic NPs and the replacement of conventional antibiotics with alternative antimicrobial agents to combat MDR pathogens.

## 2. Multimetallic NPs

Multimetallic NPs, comprising two or more different metals to form alloy or core–shell nanocomposites, have attracted considerable attention as novel materials due to their unique functionalities. The combined action of different metals and metal oxides in a chemical transformation enhances the catalytic performance of multimetallic NPs [24]. Multimetallic NPs with binary, ternary, and quaternary combinations usually have special characteristics, with enhanced chemical, optical, and catalytic properties when compared with mono- and bimetallic NPs, because of the synergistic effects between different metals [14]. Metallic NPs are classified as monometallic, bimetallic, trimetallic, quadrometallic, and so on based on the number of metallic ingredients.

### 2.1. Monometallic NPs

As the name suggests, monometallic NPs compose a single metal species, which compels for the catalytic characteristics of the nanoparticle. Based on the type of metal atom and properties, monometallic NPs are in different forms, like metallic, magnetic, transition metal, and oxide. They can be synthesized by chemical reduction and green synthetic methods, and their structure can be stabilized by various functional groups. Many studies have reported on a wide range of monometallic and metal oxide NPs, and these are used in various catalytic, medical, agricultural, active food packaging, nano-biosensor construction, industrial, and environmental applications. The order of atoms at the nanoscale, which differs from the bulk materials, is due to not only the large surface-area-to-volume ratio but also the specific electronic structure, plasmon excitation, and quantum confinement. In addition, the increased number of kinks, short-range ordering, chemical properties, and ability to store excess electrons also enhance activity. Recent studies on the antimicrobial activity of monometallic and metal oxide NPs are summarized in Table 1, highlighting the size, bacterial strains tested, mode of action, and fabrication techniques used.

### 2.2. Bimetallic NPs

Bimetallic NPs have attracted huge attention due to their modified properties, and they can be prepared in different sizes, shapes, and structure with a combination of different metals.

Extensive studies in the past decade investigated the use of bimetallic NPs as a new advancement in the field of research and as technological domains to increase efficiency. Owing to the distinct catalytic and synergistic properties between two different metals, bimetallic NPs have potential applications in more fields than their corresponding monometallic one. Depending on their physical and chemical interactions, the spatial overlapping and distribution of two atoms can lead to the formation of a core–shell or simply an alloy due to the impact of individual metals [60]. The addition of a second metal is a major technique for tuning the geometric and electronic structures of NPs to increase their catalytic activity and selectivity. The size, shape, and morphology of alloy or core–shell NPs are comparatively different from those of the individual metals, thereby creating novel opportunities for a range of biomedical applications [61]. The antimicrobial activity of bimetallic NPs has been assessed against numerous types of pathogenic bacteria, especially *E. coli*, *P. aeruginosa*, and *S. mutans*, which are mainly responsible for human epidemics. Bimetallic NPs exhibit remarkable performance compared with commonly used antibiotics and other antimicrobial treatments, as pathogens cannot develop resistance to them because they suppress the generation of biofilms and accelerate other correlated processes [62].

### 2.3. Trimetallic NPs

Trimetallic NPs have favorable properties, such as physical, chemical, and tunable properties, when compared with mono- and bimetallic NPs, which result in multiple applications for these NPs. These favorable properties are due to multifunctional or synergistic effects produced by the three metals present in the same system [20]. The addition of a third metal or metal oxide into the composite supposedly generates a combinatorial effect and introduces several possibilities for different morphologies, structures, and chemical compositions to improve catalytic activity, selectivity, and specific performance [63]. Trimetallic NPs exhibit improved reactivity because of the electronic and synergistic effects of the different elements and the geometric arrangements of the metal surrounding the absorbing atoms. At this coherence, the properties of the materials are altered due to electron transfer effects, lattice mismatching, and surface rearrangement [64]. Consequently, trimetallic NPs, such as Fe/Co/Ni supported on multiwalled carbon nanotubes (MWCNTs), show efficient and enhanced bifunctional performance for the oxygen reduction and oxygen evolution reactions [65]. Similarly, Cu/Au/Pt, with high catalytic activity and excellent killing performance for biosensing and cancer theranostics [66], and Pd/Cu/Au, as excellent temperature sensors and fluorescence detectors of H_2_O_2_ and glucose [67], have attracted attention as promising catalysts. Hence, trimetallic NPs exhibit potent antibacterial activity and have been found to be more effective agents than bimetallic and monometallic NPs, even at lower metal concentrations.

### 2.4. Quadrometallic NPs

Higher-order quadrometallic NPs are made from four different metals for various applications with different fabrication methods. Recently, solid-state dewetting of Ag/Pt/Au/Pd quadrometallic NPs on sapphire has been prepared successfully with tunable localized surface plasmon resonance [68]. Similarly, Ag/Cu/Pt/Pd quadrometallic NPs prepared by seed-mediated growth, where small Pt and Pd NPs were attached on the surface of AgCu Janus bimetallic NPs as seeds in an aqueous solution [69], and heterostructured Pt/Pd/Rh/Au tetrahexahedral multimetallic NPs were synthesized through alloying/dealloying with Bi in a tube furnace [70]. Moreover, to date, there has been no investigation into the antimicrobial properties of quadrometallic and multimetallic NPs. Table 2 summarizes bi- and trimetallic NPs used for antibacterial activity with an array of sizes, strains tested, mechanisms, and synthetic methods.

## 3. Properties of Multimetallic NPs

The properties of multimetallic NPs depend on the atomic structure, thickness, composition, shape, surface morphology, and stability of the core and shell and the disorder of alloy NPs. Furthermore, the percentage of atoms on the surface of a substance or material becomes more important for these NPs. Their enhanced surface area in contrast to their bulk metal counterparts mainly influences the elemental properties and active antibacterial properties of multimetallic NPs. Many studies have shown that tri- or multimetallic NPs are more active than mono- and bimetallic NPs made with the same metal. The catalytic properties of tri- or multimetallic NPs depend on the structure of the core and shell and the composition of the alloy NPs. Our previous studies have shown strong correlations between the number of different metals in NPs used as nanocatalysts and their activity, where trimetallic NPs show higher catalytic activities along with excellent selectivity than mono- and bimetallic NPs [92,93]. However, since different metals and metal oxides have different physicochemical properties, there will be slight parameter changes, like phase separation, agglomeration, and detachment of multimetallic NPs, which leads to poor catalytic activity and stability. On the other hand, some active research aims to initiate effective protocols, for instance, nanomaterials prepared by carbothermal shock method can enhance the overall structural and chemical stability of the multimetallic NPs [94].

Similarly, alloy nanocomposites have the special advantage of photocatalytic properties, as they possess both catalytic and plasmonic metals that absorb visible or ultraviolet light, which are released as energy to promote catalysis. The optical properties of multimetallic NPs depend on their size and shape, and they are strongly affected by their component metals. Surface plasmon resonance (SPR) increases when combining two or more metals that are vibrant in the visible region; however, it decreases or compresses when one metal in the combination is vibrant in the ultraviolet region. In addition, SPR sensitivity greatly depends on the size and shape of the nanocomposites. For instance, by decreasing the size of any metal NPs, the emission light position changes from the near-infrared to the ultraviolet region, but the sensitivity is increased when the shape of the NPs is a sphere, cube, or rod. Ultimately, nanocomposites can lose their SPR and become photoluminescent because of the very small size of the NPs [95]. In bi-, tri-, or multimetallic NPs, the SPR can provide information on the internal distribution of the elements. For alloyed nanocomposites, the shift in the SPR absorption is maximum and linear with the composition. Similarly, for core–shell nanocomposites, only one mode (frequency) of SPR is observed in the metal shell.

The magnetic properties of multimetallic NPs are influenced by several factors, including particle size and shape, chemical composition, morphology, crystal lattice, coordination of the particle with the surrounding matrix, and the adjacent particles [96]. The addition of a second or third metal into a nanoparticle structure enhances the optical/plasmonic and magnetic properties of the nanocomposites. Metals, such as iron, copper, nickel, and cobalt, possess good catalytic, electronic, and magnetic properties. Furthermore, the union of 3d metals with 4d or 5d metals, which display strong spin–orbit coupling, creates multimetallic NPs with large atomic magnetic moments and high magnetic anisotropy. Iron-based multimetallic NPs have been used in multidisciplinary fields, such as magnetic resonance imaging, drug delivery, cancer treatment, tumor detection, separation processes, and other biological activities with high biocompatibility, easy surface modifications, and low toxicity in living cells [97].

## 4. Synthetic Methods of Multimetallic NPs

**Figure 1:**
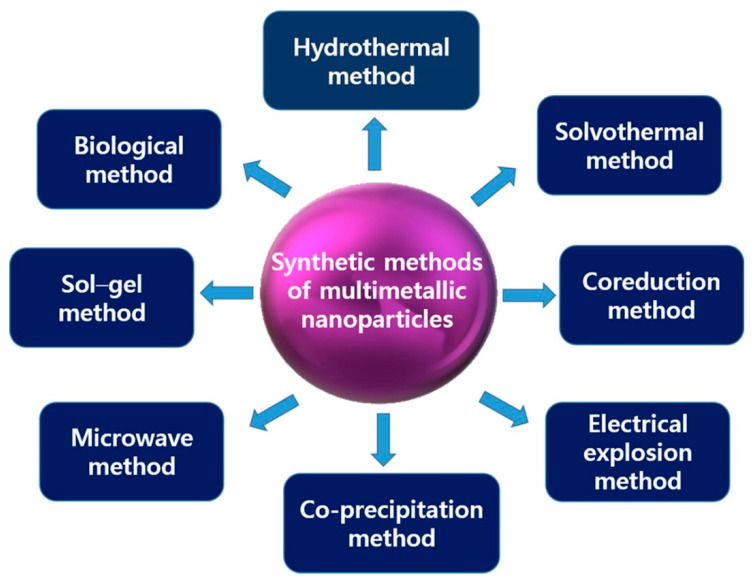
Synthetic methods for the preparation of multimetallic NPs.

Different approaches have been developed for the fabrication of multimetallic NPs, based on “top–down” and “bottom–up” techniques, with diverse procedures for functional nanomaterial alterations. The synthetic methods used to prepare multimetallic NPs are depicted in Figure 1.

### 4.1. Hydrothermal/Solvothermal Method

Hydrothermal and solvothermal techniques are the most important methods for the synthesis of various kinds of monodispersed and highly homogeneous nanomaterials. Using these methods, nanomaterials are fabricated using a typical wet-chemical approach, with high pressure and temperature, in aqueous solvents that dissolve and recover the materials. All the reactants are dissolved in an autoclave with a suitable solvent under low or high pressure and temperature conditions depending on the desired composition, crystal structure, size, and shape of the nanomaterials. The main advantage of this approach is that, with high vapor pressures and minimal loss of nanomaterials, the procedure is well controlled through liquid-phase or multiphase chemical reactions. Its disadvantages include the use of expensive equipment and high temperatures. Many bimetallic NPs, such as Ni–Fe, co-doped Zn1−xCoxMn_2_O, and NiFe_2_O_4_ NPs, have been designed using the hydrothermal method [98,99,100]. Commonly, the hydrothermal process involves the use of solvent and surfactant; however, the surfactant-free synthesis of multimetallic NPs on an electrode surface was recently reported successfully [101].

### 4.2. Coreduction Method

Coreduction is a simple method to synthesize mono-, bi-, and trimetallic NPs and can be used to produce multimetallic NPs. The two metal precursors are first dissolved in a suitable solvent along with the stabilizing agent, and the transitional metals exist in their ionic states. The reducing agent is then added to convert them into zerovalent states; however, due to their lower reduction ability, light transitional metals undergo less reduction. These mild transition metals undergo oxidation very quickly when present in their zerovalent states and are, therefore, unstable. These metals play a prominent role in catalysis, and several methods have been developed to produce transition metal NPs. The coreduction method has moderate reaction conditions when compared with thermal decomposition. The reaction occurs under air atmosphere and low temperatures, and no toxic organic solvents are used [102]. Recently, mesoporous PtPdNi alloy NPs have been synthesized by this method using three metal precursors under constant sonication [103].

### 4.3. Electrical Explosion of Wires

The electrical explosion of wires (EEW) plays an important role in several applications, such as nanoparticle production, wire-array Z-pinch, exploding wire detonators, material property investigation under the most extreme conditions, high-temperature photography, and shockwave technology for promoting fossil energy [103]. EEW is one of the most promising technologies for the synthesis of metal NPs and is based on the increased activity generated when passing a pulse current with high density across a metal wire. During the EEW procedure, a capacitor bank releases a high current flowing through a desired metal wire over a short period. The amount of energy released is much larger than the sublimation energy of the metal wire, which ultimately leads to superheating, evaporation, dispersal of the wire material into the surrounding medium, and finally, formation of NPs by condensation of the vapor [104]. Recently, antimicrobial ZnxMe(100-x)/O nanocomposites have been designed by electrical explosion of a two-wire method in an oxygen-containing atmosphere [105].

### 4.4. Coprecipitation Method

Coprecipitation is one of the simplest and most widely used methods for the synthesis of NPs in various forms, such as hydroxides, oxides, sulfides, and carbonates, with controlled sizes and magnetic properties. In this method, aqueous salt solutions, such as nitrates or chlorides, precipitate the oxo-hydroxide under an inert atmosphere. Once the solution reaches a critical concentration, a short nucleation burst occurs, followed by a subsequent growth phase. Magnetite NPs are synthesized using different bases, such as KOH, NaOH, at (C_2_H_5_)_4_NOH, at room temperature, depending on the desired crystal size, and the ratio of agglomeration promotes the formation of mesoporous structures. This chemical method is suitable for the fabrication of NPs because it does not require high temperature or pressure, and impurities are eliminated by washing and filtration. Recently, CuO-NiO-ZnO trimetallic oxide NPs have been synthesized using a coprecipitation method [90].

### 4.5. Microwave Method

The microwave heating technique is a simple, fast, reliable, versatile, and widely accepted method for the production of various nanocomposites with controlled sizes. It has a fast reaction rate and a short reaction time and high selectivity and yield when compared with conventional heating. The mechanism involved in this method affords uniform internal heating, facile nucleation, crystallization, dipolar polarization, and ionic conduction due to the force of dielectric heating. It has been found that the size and morphology of the nanocatalyst can be more easily controlled using the microwave method, when compared with conventionally prepared catalysts. Other advantages include fast ramping from temperatures of 150–250 °C in less than 1 min, compared with nearly 30 min with conventional heating methods. The microwave heating method has been employed for the preparation of trimetallic colloidal Au-Pt-Ag and Au-Pd-Pt nanocomposites using 160–800 W [106].

### 4.6. Sol–Gel Method

The sol–gel process is an effective method to produce metal oxide NPs at low temperatures compared with other physical and chemical methods. As the name suggests, a solution is transformed into a gel by a process based on the condensation reaction and hydrolysis of organometallic compounds in alcoholic solutions. The sol–gel method has excellent potential to control the reaction kinetics and the bulk and surface properties of the oxides. Moreover, it allows customizable microstructure, the addition of several functional groups, and ease of compositional modifications. The sol–gel method has been used to fabricate a variety of metal oxide NPs and bi- and trimetallic oxide nanocomposites. For instance, titanium and vanadium trimetallic nanocomposites have been synthesized with three other metals by the sol–gel technique using ammonia and hydrazine as reducing agents [107].

### 4.7. Biological Methods

Recently, green synthetic nanoparticle production methods have been developed based on biological sources, such as plant leaves, root tissues, algae, bacteria, yeast, fungi, and industrial and agricultural wastes. These methods have also been used to fabricate bi- and trimetallic alloy nanocomposites. In plants, secondary metabolites, such as flavonoids, alkaloids, terpenoids, heterocyclic compounds, polysaccharides, organic acids, proteins, and vitamins have been used as sources. In the case of microorganisms, sources, such as reductase enzymes, proteins, metal-resistant genes, and organic materials, have been used. Agricultural wastes, such as fruit peels, wild weeds, unwanted plants, burned herbs, and shrubs, are used as biological sources. In addition, industrial wastes, such as rice husks, eggshells, timber dust, and sugarcane bagasse, are used for the reduction of metal salts into NPs. Recently, Au/ZnO/Ag trimetallic nanocomposites have been synthesized using a green method that involves the use of an extract from *Melilotus officinalis* [108].

## 5. Antimicrobial Activity of Multimetallic NPs

The antimicrobial efficacies of metal and metal oxide NPs have been tested against various types of antimicrobial-susceptible and antimicrobial-resistant pathogenic strains. Pathogenic bacteria, including *E. coli*, *P. aeruginosa*, *S. mutans*, and *S. aureus*, are the main agents responsible for many infectious diseases. These pathogenic bacteria become a major challenge in the health-care system and pose a serious threat to human health. Metal and metal oxide NPs, especially multimetallics, such as bi-, tri-, and quadrometallic nanocomposites, show improved performance when compared with conventional antibiotics, as pathogens cannot develop resistance to these NPs because the nanocomposites affect biofilm formation and other associated processes. In comparison with monometallic nanomaterials, multicomponent metal nanocomposites have multiple functionalities and exhibit enhanced and cumulative properties due to the various synergistic effects of the individual components. Moreover, the catalytic and optical properties of multimetallic NPs can be successfully controlled by modifying their structure, morphology, and chemical composition. Hence, they have attracted considerable attention due to their wide range of applications in medical, sensing, and catalytic fields.

Numerous studies have demonstrated the antimicrobial activities of mono- and bimetallic NPs, as shown in Table 1 and Table 2; however, very few have reported on trimetallic NPs, as shown in Table 2. Quadro- and multimetallic nanocomposites are at the initial stages of development and are still limited in types suitable for other applications. The effectiveness of the resultant antimicrobial activities is mainly influenced by two important parameters: the type of material (precursor) used to prepare the NPs and the particle size. Generally, nanomaterials have unique properties when compared with bulk materials, and they often change dramatically with nanoscale ingredients because the surface-to-volume ratio of the NPs increases significantly with a decrease in particle size. Additionally, at nanometer dimensions, the atomic fraction of the molecule increases significantly at the surface, consequently enhancing some properties of the particles, such as dissolution rate, catalytic activity, heat treatment, and mass transfer [109]. The nanomaterials used to fight against infectious pathogens comprise mainly transition metals and metal oxides because of their special characteristics, including decreased particle size, increased surface-to-volume ratio, hydrophobic interactions, nanoparticle stability, and electrostatic attraction. These properties allow bactericidal activity and microbiostatic activity against both Gram-positive and Gram-negative bacteria.

The bactericidal mechanism of action of nanocomposites is normally described as metal ion release and oxidative stress induction, but nonoxidative mechanisms may also occur simultaneously. Moreover, the production of ROS interrupts the antioxidant defense system and causes mechanical damage to the cell membrane. In addition, the majority of recent studies have described the mechanism of action of nanocomposites as follows: adhesion to microbial cells and cell wall destruction by physical contact, leading to the generation of ROS, particle penetration into the cell, damage to proteins and DNA, oxidative stress, and facilitation of internalization. The positively charged surfaces of metal and metal oxide nanocomposites promote their binding to negatively charged bacterial surfaces, which may result in the strengthening of their bactericidal effect. In addition to size, different formulations with different particle shapes and surface charges may also influence the intrinsic properties of the NPs and potentially influence their antibacterial activity. The different morphologies, crystal growth habits, and increased lattice constants of multimetallic NPs may enhance their antibacterial activity. Multimetal and metal oxide nanocomposites have been widely studied as alternative antimicrobial agents because of the favorable synergistic effects of their individual components.

## 6. Antibacterial Mechanisms of Multimetallic NPs

**Figure 2:**
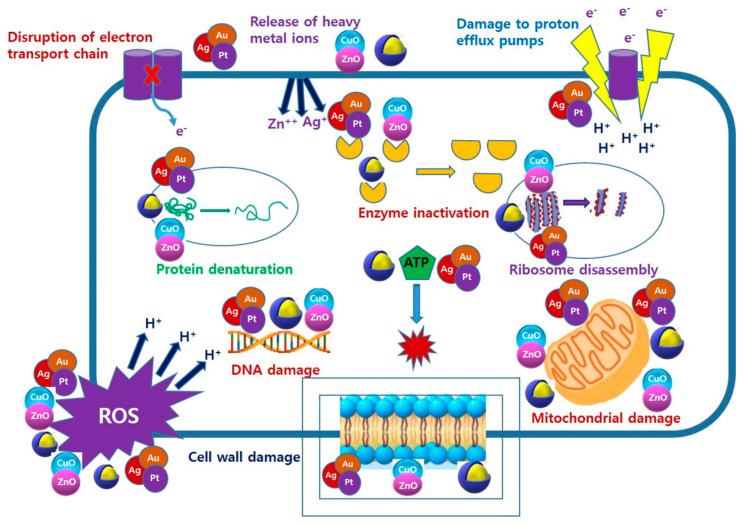
Antibacterial mechanism of multimetallic NPs.

The mode of action of bacterial destruction depends on the type of NP. Metals, metal oxides, and their nanocomposites bind to the cell wall of bacteria and form membrane-penetrating pores due to the deposition of nanomaterials on the bacterial cell surface, which causes the formation of free radicals that are able to destroy the cell membrane. In addition, ions released from the nanomaterials inhibit the production of enzymes and increase the production of ROS, which in turn affects DNA transcription [110]. The mode of action of multimetallic NPs is summarized in Figure 2.

### 6.1. Disruption of Cell Membrane

Metal- and metal oxide-based nanocomposites can damage the bacterial cell membrane by electrostatically binding to the cell wall and releasing metallic ions. The difference in charge between bacterial membranes (negative charge) and nanocomposites (positive charge) induces an electrostatic attraction and changes the permeability of the cell membrane. Hence, the disturbance of the bacterial membrane integrity is an effective mechanism of action. Based on the structure of the cell wall, bacteria are divided into two groups: Gram-positive bacteria, which have many layers of thick peptidoglycan in their cell wall, and Gram-negative bacteria, which have a cell wall consisting of a thin peptidoglycan layer with an additional outer membrane of lipopolysaccharide. The negatively charged properties of both Gram-positive and Gram-negative bacterial cell walls affect the interactions between the cell wall and nanoparticle ions.

Metal and metal oxide NPs exhibit higher antibacterial activity against Gram-negative bacteria than Gram-positive bacteria [111] because the negative charge of lipopolysaccharides allows the adherence of NPs to Gram-negative bacterial cell walls. Thus, a high NP-binding capability on these negative anionic areas may increase toxicity due to high NP concentrations. A layer of lipopolysaccharides covers the cell wall of the Gram-negative bacterium, *E. coli*, and peptidoglycans, whereas the cell wall of the Gram-positive bacterium, *S. aureus*, consists of a peptidoglycan layer and is much thicker than the Gram-negative bacterial cell wall. The difference in composition, structure, and thickness of the cell wall allows NPs to penetrate, resulting in less inhibition of *S. aureus* than *E. coli*, which shows significant inhibition even at low antibiotic concentrations. Using this approach, there is a clear correlation between the concentration of the NPs and the different classes of bacteria being treated due to differences in the chemical and structural organization of the cell wall.

### 6.2. Formation of ROS

ROS are highly reactive metabolic products with strong positive redox potential. They are produced in numerous cells by two cellular organelles, the endoplasmic reticulum and mitochondria. The production of ROS is an alternative mechanism by which nanomaterials kill bacteria. Different types of ROS, namely, the superoxide radical (O_2_^−^), singlet oxygen (^1^O_2_), hydrogen peroxide (H_2_O_2_), and the hydroxyl radical (OH), exhibit different levels of activity. Superoxide, an anion radical (O_2_^−^), is a potent oxidizing agent that is highly reactive with water and is produced mainly in the thylakoid-localized photosystem I (PSI) by one electron, as well as in other cellular compartments. Singlet oxygen is a strong reagent that facilitates undesirable oxidation inside the cell and causes severe damage to various molecules of biological importance. The production of singlet oxygen leads to peroxidation of cellular constituents, such as proteins and lipids. ROS react with H ions to produce hydrogen peroxide, which more easily penetrates cell membranes and destroys various cellular organelles, resulting in bacterial death. Hydroxyl radicals are fatal to pathogenic bacteria; however, some hydroxyl radicals that are negatively charged cannot easily penetrate the negatively charged cell membrane. The diffusion of metal ions from metal oxide NPs generates a large number of hydroxyl radicals due to the decomposition of bacterial cells. ROS are formed when oxygen enters reduction states and is converted into free radicals, such as superoxides and peroxides, instead of water. However, some pathogens can fight back in response to ROS by producing superoxide dismutase enzymes. ROS induce acute oxidative stress, inhibit enzymes, and cause damage to lipids, proteins, and DNA/RNA. Gold, magnesium oxide, and zinc oxide promote ROS formation through increased catalytic activity by producing H_2_O_2_ from glucose oxidase [112].

### 6.3. Dysfunction of Cytosolic Proteins

Protein dysfunction is another mode of action by which multimetallic NPs induce their antimicrobial response by binding to cytosolic proteins, such as DNA and enzymes. The metal-ion-catalyzed oxidation of the amino acid side-chain leads to the formation of protein-bound carbonyls. The carboxylation of protein molecules may serve as a better marker for oxidative damage to proteins. However, in the case of enzymes, carboxylation results in the loss of catalytic activity and stimulates protein degradation [113]. Silver and copper NPs inhibit DNA replication, cell division, and DNA degradation [114,115], whereas gold NPs interact with DNA within the cell by upregulating genes [111], leading to decreased membrane integrity and the accumulation of ROS within the cytosol. Many studies have reported that when bacterial cells are exposed to nanomaterials, the physical attachment of NPs leads to nuclear DNA fragmentation and damage due to the high affinity of metal ions for phosphate, which is highly abundant in DNA molecules [116].

## 7. Synergistic Effects of Multimetallic NPs

A synergistic effect is a process in which biological structures or chemical substances combine to create an effect that is greater than either one of them could have caused alone. Multimetallic NPs have attracted more attention than monometallic NPs from researchers in different fields, such as physics, biology, and medicine, because of their widespread applications in catalysis, sensing, and medical fields. Multimetallic NPs are obtained by incorporating three or more metal elements with various nanostructures and different properties into single nanomaterials called alloys. These multifunctional nanomaterials exhibit advanced properties with novel functions due to the synergistic effects of different elements. The continuous modulation of the electronic structure of multimetallic NPs enhances their catalytic performance because of the various binding forces acting on electrons between different metal atoms [117]. Hence, the synergistic effects of multimetallic nanocatalysts play a prominent role in the field of heterogeneous catalysis, especially in oxidation and reduction reactions [118]. Multimetallic NPs also show synergistic effects by the combination of two or more metals with an atomic ratio of various metals, ultimately achieving high catalytic efficiency, including high selectivity and catalytic activity [119].

The synergistic effect of nanocatalysts is influenced by geometric parameters, local strain, electronic states, and successful coordination at the surface [66]. In addition, multimetallic NPs modify the electronic structure of metals, which permits the tuning of the binding energy between nanocatalysts and reaction intermediates, thus initiating a synergistic effect that can enhance the durability and catalytic activity of the NPs [66]. In the case of tandem reactions, which require two or more catalysts, multimetallic NPs improve the catalytic properties by forming an interconnection area between two or more metals [120]. For instance, our previous studies showed that FeAgPt alloy trimetallic NPs performed better than FeAg and FePt bimetallic and Fe, Ag, and Pt monometallic NPs in reduction and decolorization reactions, with excellent selectivity and activity [92]. Similarly, AuFeAg hybrid NPs, a convenient green catalyst, showed greater catalytic activity than FeAu and FeAg bimetallic NPs and Au, Fe, and Ag monometallic NPs in the preparation of α,β- and β,β-dichloroenone from diazodicarbonyl and oxalyl chloride [93]. Recently, FeCoNi mixed oxide NPs supported on oxidized MWCNTs were used as catalysts for oxygen reduction and oxygen evolution reactions, and they demonstrated enhanced selectivity with respect to the reduction of O_2_ to OH− when compared with FeNi, FeCo, and CoNi bimetallic catalysts, thus suggesting synergistic effects among the metal oxide elements [65]. Similarly, the antimicrobial activity of trimetallic NPs shows synergistic effects when compared with bi- and trimetallic NPs, as shown in Table 2 [63,88,89,90,91]. Therefore, greater attention has been paid to synergistic interactions or combinatorial therapy as an alternative strategy to combat antibiotic-resistant infectious pathogens.

## 8. Toxicity of Multimetallic NPs

In recent decades, various efforts have been made to evaluate the toxicological impact and possible hazards and risks of different NPs on human health and the environment. Multimetal and metal oxide NPs have great potential to manage several diseases and infection by resistant bacterial strains because of their special physicochemical properties and small size, thus enabling the particles to be ingested or inhaled or to penetrate through the skin more readily. However, there is increasing concern about the safety of prolonged exposure to these NPs before their application in various fields and their large-scale production. The range of toxic effects depends on the nature of the metal and metal oxide NPs and their surface functional groups. The in vitro evaluation of different types of NP interactions with living cells of humans, animals, plants, and aquatic organisms has already been conducted. However, major issues, such as the behavior of NPs inside the cells, their tissue penetration, and the metabolic and immunological responses they induce are often ignored.

Predictions of toxicity are certainly useful for understanding the mechanisms of nanotoxicology, interactions of nanomaterials with biological systems, and careful assessment of nanomaterial properties. When evaluating toxicity, it is important to note that different organisms have different sensitivities to NPs, and differences in NP concentration and solubility, the presence of additives, and the synthetic methods used are clearly accounted for in some reported results. Metal NPs, including Au and Ag NPs, and metal oxide NPs, including MgO, CuO, Fe_3_O_4_, ZnO, and TiO_2_ NPs, are the most promising antimicrobial agents, with low toxicity against human cells [121]; however, more research is needed to clarify the mechanisms of NP migration into the human body. The migration of NPs into the human body depends on the size, structure, solubility, and chemical composition of the NPs, and because of their small size, NPs can pass through different organs and settle in the central nervous system to stimulate an immune system response. The surface chemistry of NPs is another factor that determines their cytotoxicity and their effects on biological systems due to the resulting charge, roughness, and hydrophobic or hydrophilic nature [122]. In several studies, metal and metal oxide NPs have been shown to be toxic at higher concentrations in human fibroblasts, kidneys, liver cells, and macrophages [123].

## 9. Concluding Remarks and Future Perspectives

The use of alternative strategies to combat MDR pathogens is warranted because of their constantly increasing resistance to current antibiotics. Pathogens usually develop resistance to antibiotics as they are overused and misused in husbandry practices, with no effective management. Nanotechnology has been applied in almost every field of science, including materials science, physics, chemistry, computer science, biology, and engineering, over the last decade. In recent years, nanotechnology has also been applied to human health as a promising candidate for the treatment of infectious bacterial diseases. Nanomaterials possess superior properties due to their optical, electrical, mechanical, and magnetic properties, thermal stability, small dimensions, and high surface-to-volume ratio. Thus, nanomaterials are considered an alternative therapeutic option to control bacterial infections because of their size, shape, solubility, surface charge, stability, and surface coatings.

As discussed previously, various studies have been performed on a wide range of monometallic and metal oxide NPs, and these have demonstrated potential antimicrobial activities against MDR pathogens. Despite these potential advantages, very few studies have reported the synergistic effects of bi-, tri-, and quadrometallic and metal oxide nanocomposites. Multimetallic NPs, such as bi-, tri-, and quadrometallic NPs, have attracted great interest because of their favorable characteristics as advanced materials and their enhanced catalytic properties when compared with monometallic NPs. The combination of two or more nanomaterials to form a single system can enhance their special optical, catalytic, electronic, and magnetic properties. This is expected to bring combinatorial effects, such as alterations of the electronic structure and a reduction in the lattice distance. In addition, tuning or altering the size, shape, elemental composition, internal structure, and surface modification of nanomaterials can result in the formation of nano-alloys, core–shell structures, and heterodimers with enhanced catalytic performance. Furthermore, the use of multimetallic NPs as hybrid materials also provides specific plasmon excitation, an increased number of kinks, changes in the electronic and magnetic properties, short-range ordering, altered chemical properties, improvements in the overall electronic charge shift, and the ability to store more electrons.

The exact contribution of multimetallic NPs to the treatment of MDR pathogens has not yet been elucidated, and further research is needed to identify alternative antimicrobial agents by modulating the shape, size, and surface chemistry of multimetallic NPs. Thus, multimetallic NPs are a potential source of alternative antimicrobial agents and may play a significant and synergistic role in the near future. The present paper discussed findings on the in vitro antimicrobial activities of nanomaterials; however, further studies are essential to assess the synergistic effects of bi-, tri-, and multimetallic NPs in vivo. Furthermore, careful selection of the nanomaterial type, the appropriate dosage, and the choice of application is crucial for beneficial outcomes, because the majority of nanomaterials are metallic and may lead to potential uptake and accumulation. In addition, the management of environmental issues, health and safety aspects, risk assessment, potential toxicity, and hazards must be considered before introducing them as safe and effective antimicrobial agents. In conclusion, a broad understanding of these new and creative therapeutic strategies is essential as they may serve as alternatives to conventional antibiotics. The present study suggests that multimetallic NPs and their composites can be utilized to develop more effective antimicrobial agents in the near future to prevent the emergence and spread of bacterial resistance to conventional antibiotics, and they may play an important role in many medical applications.

## Figures and Tables

**Table 1:** Antimicrobial activity of monometallic and metal oxide nanoparticles (NPs).

NPs	Size (nm)	Bacteria	Mode of Action	Synthesis	Ref.
Ag	10	*V. natriegens*	DNA damage and cell membrane rupture by reactive oxygen species (ROS)	Green catalysis	[25]
Au	20	*S. pneumoniae*	Cell lysis	Chemical reduction	[26]
Pd	13–18	*S. aureus, S. pyrogens, B. subtilis*	Cell membrane destruction and apoptosis	Biosynthesis (plant)	[27]
Ga	305	*M. tuberculosis*	Reduction of the growth of mycobacterium	Homogenizer	[28]
Cu	15–25	*S. aureus, B. subtilis*	Synergistic effects of organic functional groups	Biosynthesis (plant)	[29]
Pt	2–5	*E. coli, A. hydrophila*	Decrease in the bacterial cell viability and ROS generation	Chemical reduction	[30]
Si	90–100	*S. aureus, P. aeruginosa*	Mechanical damage of the bacterial membrane	Laser ablation	[31]
Se	117	*Klebsiella* sp.	Production of ROS, disruption of the phospholipid bilayer	Biosynthesis (plant)	[32]
	55.9	*B. subtilis, E. coli*	Ionic interaction between NPs and bacteria-caused cell damage	Biosynthesis (plant)	[33]
	85	*E. coli, S. aureus*	Cell membrane damage due to action of ROS	Laser ablation	[34]
Ni	60	*P. aeruginosa*	Cell membrane destruction	Biosynthesis (plant)	[35]
Mn	50–100	*S. aureus, E. coli*	Inactivation of proteins and decrease in the membrane permeability	Biosynthesis (plant)	[36]
Fe	474	*E. coli.*	Attraction between negatively charged cell membrane and NPs	Biosynthesis (plant)	[37]
Bi	40	*B. anthracis, C. jejuni, E. coli, M. arginini*	Inhibition of protein synthesis	Chemical condensation	[38]
Ag_2_O	10–60	*S. mutans, L. acidophilus*	Penetration of the cells and hindrance of the growth of the pathogen	Biosynthesis (plant)	[39]
CuO	60	*B. cereus*	Disturbance of various biochemical processes when copper ions invade inside the cells	Biosynthesis (plant)	[40]
ZnO	30	*A. baumannii*	Increase in the production of ROS	Sol–gel and biosynthesis	[41]
TiO_2_	9.2	*E. coli*	Decomposition of outer cell membrane by ROS, primarily hydroxyl radicals (OH·)	Biosynthesis (plant)	[42]
NiO	40–100	*B. subtilis, E. coli*	Induction of membrane damage by oxidative stress created at the NiO NP interface	Hydrothermal	[43]
Fe_3_O_4_	25–40	*S. aureus, E. coli, S. dysentery*	Cellular enzyme deactivation and disruption in plasma membrane permeability	Coprecipitation	[44]
α-Fe_2_O_3_	16	*B. subtilis, S. aureus, E. coli, K. pneumonia*	Desorption of membrane by the generated free radicals, including O_2_**·** and OH·	Biosynthesis (plant)	[45]
CaO	58	*E. coli, S. aureus, K. pneumonia*	Cell membrane destruction	Biosynthesis (plant)	[46]
MgO	27	*Bacillus* sp., *E. coli*	Destruction of cell membrane integrity resulting in leakage of intracellular materials	Ultrasonication	[47]
Al_2_O_3_	30–50	*F. oxysporum, S. typhi, A. flavus, C. violaceum*	Decomposition of bacterial outer membranes by ROS	Biosynthesis (fungi)	[48]
CeO_2_	5–20	*L. monocytogenes, E. coli, B. cereus*	ROS generation by CeO_2_ as a pro-oxidant	Precipitation	[49]
Mn_3_O_4_	130	*K. pneumonia, P. aeruginosa*	Membrane damage of bacterial cells by the easy penetration of Mn_3_O_4_ NPs	Hydrothermal	[50]
ZrO_2_	2.5	*S. mutans, S. mitis, R. dentocariosa, R. mucilaginosa*	Enhancement of the interactions between NPs and bacterial constituents	Solvothermal	[51]
Ag_2_S	65	*Phormidium* spp.	Inhibition of cell membrane by Ag_2_S NPs, resulting in harmful effects on other biological activities	Chemical reduction	[52]
ZnS	65	*Streptococcus* sp., *S. aureus, Lactobacillus* sp., *C. albicans*	Dischargement of ions, which react with the thiol groups in the proteins present on the cell membrane	Biosynthesis (bacteria)	[53]
CdS	25	*Streptococcus* sp., *S. aureus, Lactobacillus* sp., *C. albicans*	Impregnation and surrounding the bacterial cells by CdS NPs	Biosynthesis (bacteria)	[53]
FeS	35	*S. aureus, E. coli, E. faecalis*	NP internalization through the fine cell membrane	Hydrothermal	[54]
Mn-MOF	˗	*E. coli, E. faecalis, S. aureus, P. aeruginosa*	Peptide–nalidixic acid conjugate formation	Mechanochemical	[55]
Mg-MOF	˗	*E. coli, E. faecalis, S. aureus, P. aeruginosa*	Peptide–nalidixic acid conjugate formation	Mechanochemical	[55]
Ag-MOF	˗	*S. aureus*	High stability in water and the existence of Ag ion	Solvothermal	[56]
Cu-MOF	˗	*S. aureus, E. coli, K. pneumonia, P. aeruginosa, S. aureus*	Attachment to the bacterial surfaces by active surface metal sites in Cu-MOF	Hydrothermal	[57]
Zn-MOF	˗	*P. aeruginosa*	Penetration inside the bacteria, causing cell damage by interaction with lipotropic acid	Solvothermal	[58]
Co-MOF	˗	*E. coli*	Strong interaction with membranes containing glycerophosphoryl moieties	Hydro-solvothermal	[59]

**Table 2:** Antimicrobial activity of bimetallic and trimetallic NPs.

NPs	Size (nm)	Bacteria	Mode of Action	Synthesis	Ref.
Ag/Au	9.7	*E. coli, S. aureus*	Increased production of ROS	Green	[71]
Ag/Cu	26	*E. coli, B. subtilis*	Permeability of copper and silver ions into the bacterial cell membrane	Biosynthesis (plant)	[72]
Au/Pt	2–10	*S. aureus, P. aeruginosa, C. albicans*	Release of Ag^+^ ions, unbalance of cell metabolism, and ROS generation	Chemical reduction	[73]
Ag/Fe	110	*S. aureus, P. aeruginosa*	Release of Ag+ ions and ROS generation	Electrical explosion	[74]
Ag/Pt	36	*E. faecalis, E. coli*	Increased production of ROS	Biosynthesis (plant)	[75]
Cu/Zn	100	*A. faecalis, S. aureus, C. freundii*	Synergistic properties of Zn^2+^ and Cu^2+^ ions together	Biosynthesis (plant)	[76]
Cu–Ni	25	*S. mutans, S. aureus, E. coli*	Strong adsorption of ions to the bacterial cells	Chemical reduction	[77]
Ag/ZnO	43	*S. aureus, P. aeruginosa*	Ag^+^ leaching from metallic silver	Photoreduction	[78]
Ag/SnO_2_	9	*B. subtilis, P. aeruginosa, E. coli*	Synergistic properties of Ag and SnO	Biosynthesis (plant)	[79]
Cu/FeO_2_	32.4	*B. subtilis, X. campestris*	DNA damage induced by NPs	Hydrothermal	[80]
Au/CuS	2–5	*B. anthracis*	Disordered and damaged membranes	Seeded	[81]
Fe_3_S_4_/Ag	226	*S. aureus, E. coli*	Release of Ag^+^ions and ROS generation	Solvothermal	[82]
MgO/ZnO	10	*P. mirabilis*	Alteration of cell membrane activity, ion release, and ROS production	Precipitation	[83]
CuO/ZnO,	50 and 82	*E. coli, S. aureus*	Electrostatic interaction causing to change membrane permeability on account of depolarization	Electrical explosion	[84]
CuO/Ag	20–100	*L. innocua, S. enteritidis*	Binding of the ions released by μCuO/nAg to the thiol groups of many enzymes in cell membrane	Hydrothermal	[85]
Fe_3_O_4_/ZnO,	200–800	*S. aureus, E. coli*	Membrane stress, disrupting and damaging cell membrane	Coprecipitation	[86]
CeO_2_/FeO_2_	40 and 25	*P. aeruginosa*	Combination of NPs with antibiotic ciprofloxacin, causing inhibitory effect on bacterial growth and biofilm formation	Hydrothermal	[87]
Cu/Zn/Fe	42	*E. faecalis, E. coli*	Interruption of cellular processes by released ions, which can cross cell membranes	Chemical reduction	[88]
Au/Pt/Ag	20–40	*E. coli, S. typhi, E. faecalis*	Generation of ROS	Microwave	[89]
Cu/Cr/Ni	100–200	*E. coli, S. aureus*	Antibacterial activity of trimetallic NPs in comparison with pure metals	Biosynthesis (plant)	[63]
CuO/NiO/ZnO	7	*S. aureus, E. coli*	Ruptured and cracked bacterial cells by the release of intracellular components	Coprecipitation	[90]
Ag/ZnO/TiO_2_	60–170	*E. coli*	Reduction in the bandgap energy by increasing the e^−^ & h^+^ charge separation time	Sol–gel	[91]

## Data Availability

Not applicable.
